# Genetic Variation in a MicroRNA-502 Minding Site in *SET8* Gene Confers Clinical Outcome of Non-Small Cell Lung Cancer in a Chinese Population

**DOI:** 10.1371/journal.pone.0077024

**Published:** 2013-10-11

**Authors:** Jiali Xu, Zhiqiang Yin, Wen Gao, Lingxiang Liu, Yongmei Yin, Ping Liu, Yongqian Shu

**Affiliations:** 1 Department of Oncology, The First Affiliated Hospital of Nanjing Medical University, Nanjing, China; 2 Department of Dermatology, The First Affiliated Hospital of Nanjing Medical University, Nanjing, China; IPO, Inst Port Oncology, Portugal

## Abstract

**Background:**

Genetic variants may influence microRNA-target interaction through modulate their binding affinity, creating or destroying miRNA-binding sites. SET8, a member of the SET domain-containing methyltransferase, has been implicated in a variety array of biological processes.

**Methods:**

Using Taqman assay, we genotyped a polymorphism rs16917496 T>C within the miR-502 binding site in the 3′-untranslated region of the *SET8* gene in 576 non-small cell lung cancer (NSCLC) patients. Functions of rs16917496 were investigated using luciferase activity assay and validated by immunostaining.

**Results:**

Log-rank test and cox regression indicated that the CC genotype was associated with a longer survival and a reduced risk of death for NSCLC [58.0 vs. 41.0 months, *P* = 0.031; hazard ratio = 0.44, 95% confidential interval: 0.26–0.74]. Further stepwise regression analysis suggested rs16917496 was an independently favorable factor for prognosis and the protective effect more prominent in never smokers, patients without diabetes and patients who received chemotherapy. A significant interaction was observed between rs16917496 and smoking status in relation to NSCLC survival (*P*<0.001). Luciferase activity assay showed a lower expression level for C allele as compared with T allele, and the miR-502 had an effect on modulation of *SET8* gene *in vitro*. The CC genotype was associated with reduced SET8 protein expression based on immunostaining of 192 NSCLC tissue sample (*P* = 0.007). Lower levels of SET8 were associated with a non-significantly longer survival (55.0 vs. 43.1 months).

**Conclusion:**

Our data suggested that the rs16917496 T>C located at miR-502 binding site contributes to NSCLC survival by altering SET8 expression through modulating miRNA-target interaction.

## Introduction

Lung cancer continues to be the leading cause of cancer-related death worldwide, due to its high incidence, malignant behavior and lack of major advancements in treatment strategy. Non-small cell lung cancer (NSCLC) accounts for about 80% of all cases of lung cancer, with less than 15% of patients surviving beyond 5 years [1], [2]. The discovery and application of specific prognostic biomarkers in addition to the standard tumor, lymph node, and metastasis (TNM) staging system may improve the medical care of patients with NSCLC [3]. Despite intense efforts [4], [5], [6], there is still a lack of specific biomarkers for lung cancer prognosis prediction. An ideal biomarker should be easy to detect, stable and reproducible. Genetic variations in cancer patients might serve as prognostic markers of clinical outcome.

MicroRNAs (miRNAs) are a class of small (∼20–22 nt) non-coding RNA molecules that regulate gene expression through binding the 3′-untranslated region (3′UTR) of targeted mRNA [7]. It is estimated that about 30% of human genes are transcriptional or posttranscriptional regulated by miRNAs. As a result, miRNAs are involved in crucial biological processes, including development, differentiation, apoptosis and proliferation [8], [9]. Recent studies have demonstrated the dysregulated miRNA expression patterns in diverse cancers, indicating important roles of miRNAs in the initiation, progression and metastasis of human cancer [10], [11]. MiRNA expression profiles and specific miRNAs have been shown to associate with survival of a variety of cancers, including lung cancer [12], [13], [14]. Single-nucleotide polymorphisms (SNPs) in pre-miRNA or mature miRNA sequences and miRNA-binding sites may modulated the miRNA-target interactions through altering miRNA expression, maturation, destroying or creating the miRNA-binding sites, resulting in the deregulation of target gene expression [15], [16], [17]. This kind of polymorphisms have been implicated in cancer susceptibility, chemotherapy sensitivity and prognosis [16], [18], [19], [20]. Among these miRNA-binding site SNPs is the one found within the miR-502 binding site in the 3′UTR of the histone methyltransferase *SET8* gene [21].

**Figure 1:**
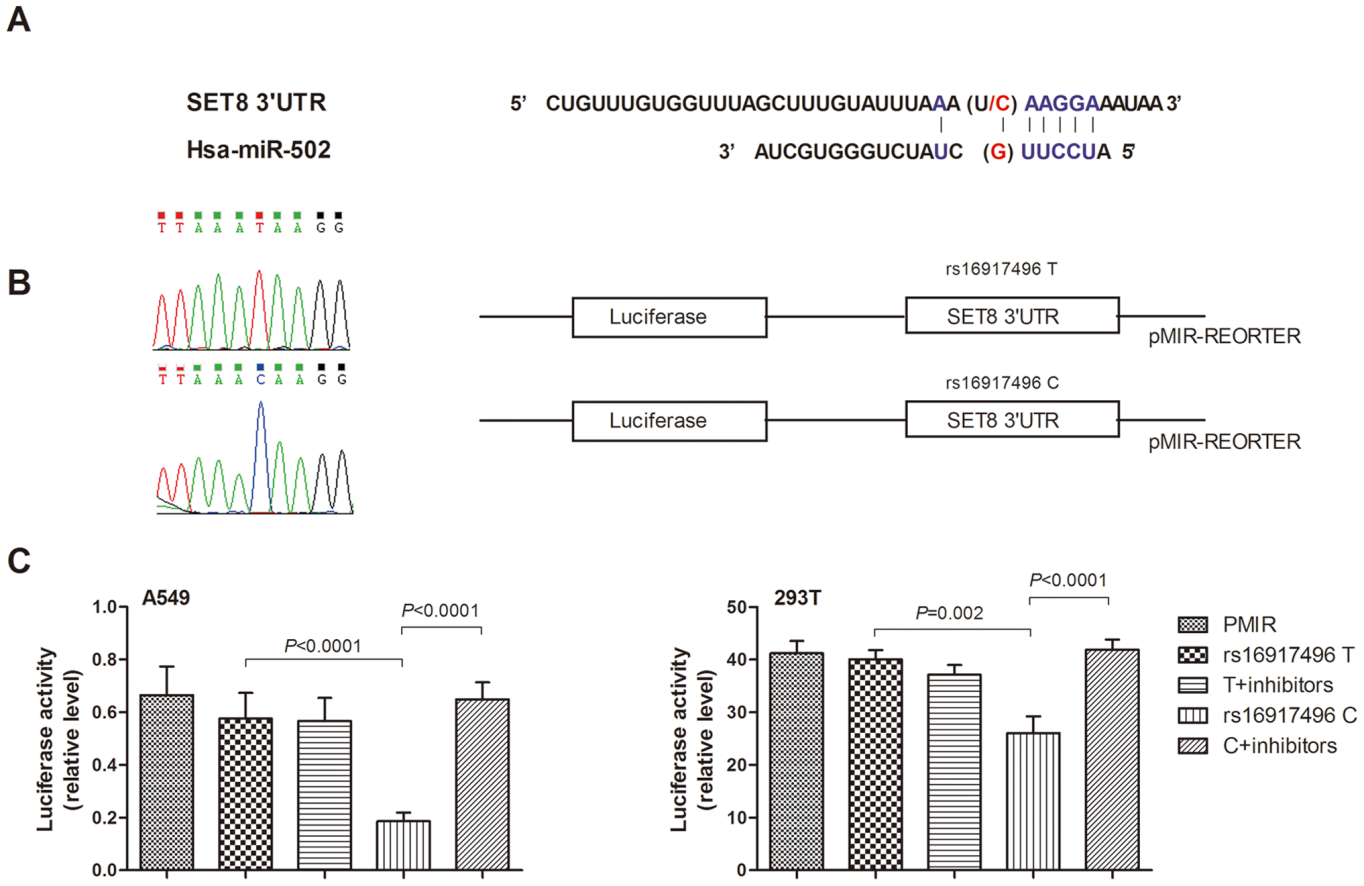
Genomic structure of *SET8*, reporter gene constructs for the 3′UTR of *SET8* and luciferase expression of the constructed plasmid in different cell lines. (A) The sequence complementarity of has-miR-502 and *SET8* 3′UTR is shown here. (B) Direct sequencing and Schematic drawing of the reporter constructs containing 1650 bp 3′UTR of *SET8* with rs16917496 T or C allele. (C) Luciferase expression of constructs containing rs16917496 T or C allele in A549 and 293T cells. Each transfection was performed with pRL-SV40 plasmids as normalized controls. *SET8* 3′UTR luciferase reporter plasmids were cotransfected with chemically synthesized mature hsa-miR-502 with or without miR-502 inhibitors in A549 and 293T cell lines. 3′UTR, 3′-untranslated region.

*SET8* (also known as PR-SET7/SETD8/KMT5A; located on chromosome 12q24.31), a member of the SET domain-containing methyltransferase family especially targeting H4K20 for monomethylation [22], [23], has been implicated in a variety array of biological processes, such as transcriptional regulation [24], [25], heterochromatin formation [26], genomic stability [27], [28], cell-cycle progression and development [28], [29], [30], [31], [32]. Recently, Shi et al. [33] demonstrate an activity for SET8 as a p53 methylatransferase and Yang et al. [34] revealed a novel role for SET8 in tumor invasion and metastasis. Previous studies suggest a polymorphism rs16917496 T>C, which is located within the miR-502 binding site in *SET8* 3′UTR (Figure 1A), modulates SET8 protein expression, and thus contributes to breast cancer and ovarian cancer susceptibility, and clinical outcome of hepatocellular carcinoma [35], [36], [37].

In this study, we genotyped rs16917496 in NSCLC patients to demonstrate that this SNP is an important genetic variant for survival prediction. We also validated that SNP rs16917496 was related to *SET8* expression through affecting miR-502 binding to *SET8* 3′UTR.

## Materials and Methods

### Ethics Statement

This study was approved by the institutional review board of Nanjing Medical University. All participants were voluntary and would complete the informed consent in written before taking part in this research.

### Study Population

All subjects were recruited from the First Affiliated Hospital of Nanjing Medical University (Jiangsu, China) between January 2004 and September 2012. All patients were newly diagnosed, histopathologically confirmed and without prior history of other cancers or previous chemo- or radiotherapy. All of the subjects were unrelated ethnic Han Chinese population. After written informed consent was obtained, a structured questionnaire on demographic data and environmental exposure history, such as age, sex and smoking consumption, was administered through face-to-face interviews by trained interviewers. Each patient donated 5-ml venous blood for genomic DNA extraction. Subjects with a low frequency (<1 cigarette per day) and duration (<1 year) of smoking were defined as nonsmokers; all others were classified as smokers. Follow-up was performed every 3 months from the time of enrollment until death or the last scheduled follow-up (last follow-up in February 2013). We selected the patients with complete follow-ups and adequate DNA sample. As a result, 576 NSCLC patients were included and genotyped in our study. The maximun follow-up time was 102 months (last follow-up in February 2013) and the medial follow-up time was 18.0 months.

### Genotyping

The genomic DNA of each subject was extracted by a routine method [38]. TaqMan allelic discrimination assay was chosen for genotyping using an ABI 7900 system (Applied Biosystems, Foster City, CA, USA). Primer and probe are: forward, TTTATGATGACAAATAATTTTCAAGTT, reverse, AATGTGAGACACAATGTCTTGATTATA and FAM-TTTATTTCCTTGTTTAAA-MGB, HEX-TTTATTTCCTTATTTAAAT-MGB. The genotyping assay included two blank (water) controls in each 384-well format and more than 10% of samples were randomly selected for repeat analysis, yielding 100% concordance.

### Construction of Reporter Plasmids

Since a significant association was later observed for rs16917496 T>C polymorphism and NSCLC survival, we constructed two reporter plasmids containing rs16917496 T or rs16917496 C allele to determine whether this polymorphism had any effect on its gene expression (Figure 1B). The T allele reporter construct was synthetic using standard DNA techniques (Invitrogen, Carlsbad, CA, USA). The product and pMIR-REPORT™ (Appied biosystems) vector with renilla and firefly luciferase gene sequences were cleaved by using *Mlu* I and *Sac* I (NEB) and then ligated by T4 DNA ligase (NEB). The C allele of rs16917496 was generated with the site-directed mutagenesis kit (Takara, Berkeley, CA, USA) with forward mutagenic primer 5′-AAAGAAgAAGGAACTAGGTCAAAAATCTGTCC-3′ and reverse mutagenic primer 5′-TAGAGCAAAAAGAACTTTTACCTCGGCATC-3′ according to the manufacturer’s protocol. All constructs used in this study were verified by directing sequencing (Figure 1B).

### RNA Interferences, Transient Transfections and Luciferase Assays

The rs16917496 T>C polymorphism located at the binding site of miR-502 (Figure 1A). So, we applied the mimic and inhibitor of this miRNA that synthesized by GenePharma (Shanghai, China) to show their effect on pMIR-SET8 reporter gene in vitro. The A549 and 293T cells were maintained in RPMI 1640 medium with 10% heat-inactivated fetal bovine serum (Gibco, Carlsbad, CA, USA) and 50 µg/ml streptomycin (Gibco). Cells were seeded into 24-well plates at 1×10^5^ cells per well and cultured at a 37°C incubator supplemented with 5% CO_2_ for 24 h. The cells were then transiently co-transfected with the *SET8* 3′UTR luciferase plasmids (different alleles) and miR-502 mimcs with or without miR-502 inhibitors using Lipofectamine 2000 according to the protocol (Invitrogen). The pRL-SV40 plasmid (Promega, Madison, WI, USA) was also transfected as a normalizing control. At 24 h after transfection, cells were collected and analyzed for luciferase activity with Dual-Luciferase Reporter Assay System (Promega). Independent triplicate experiments were done for each plasmid construct.

### Immunohistochemistry

The expression of SET8 proteins in lung cancer was detected by immunohistochemistry (IHC). Slides were prepared using a Ventana autoimmunostainer (Roche Applied Science, Mannheim, Germany) and an anti-SET8 antibody (Abcam, Cambridge, UK). Detection utilized Polymer-HRP, with 3,3-diaminobenzidine. Slides were visualized at 40× with a Nikon Eclipse microscope.

The sections were reviewed and scored by two pathologists that were blinded to the genotyping results. Controversial cases were re-evaluated jointly until a consensus was reached. For comparison of the staining results, samples were scored semi-quantitatively using a histologic score (H-score). The intensity of tumor cell nuclear immunoreactivity (1, none or weak; 2, moderate; and 3, intense) was multiplied by the percentage of positive neoplastic cells (1–100), thus obtaining values from 0 to 300. SET8 expression was categorized as high (at or above the median H-score value) or low (below the median H-score value).

### Statistical Analysis

Hardy–Weinberg equilibrium was assessed by a goodness-of-fit χ^2^ test. Overall survival (OS) was calculated as the time between the first treatment and death or the last follow-up date. Association between genotype and survival rate was estimated by the Kaplan–Meier method and log-rank test. The median survival time (MST) was calculated, and the mean time is presented when the median time could not be calculated. Cox proportional hazards models were performed to estimate the hazard ratios (HRs) and their 95% confidential intervals (CIs) for OS. The *P* value for the heterogeneity test was based on the χ^2^-based Q test. The statistical power was calculated by using the PS Software (http://biostat.mc.vanderbilt.edu/twiki/bin/view/Main/PowerSampleSize). Student’s t-test was used to compare the difference in levels of luciferase reporter gene expression. The distribution of IHC expression grades for each *SET8* genotype was compared using a χ^2^ test. All statistical analyses were performed using SPSS 18.0 software (SPSS Inc.), and *P*<0.05 in a two-side test was considered to be statistically significant.

## Results

### Characteristics of the Study Population

The demographic characteristics and clinical information of the patients and the association with OS are shown in Table 1. The median age at diagnose was 60 years (range, 29–86), and there were 380 males (66.0%) and 267 smokers (46.4%). Among these patients, 381 (66.2%) were adenocarcinomas, 166 (28.8%) were squamous cell carcinomas, and the others (29 patients, 5.0%) were large cell, undifferentiated and mixed-cell carcinomas. During the follow-up period, 206 patients died from NSCLC. Smoking status, clinical stage and surgical operation, but not chemotherapy or targeted therapy status, were significantly associated with survival time (all log-rank P<0.001). Interestingly, patients with diabetes (MST, 54.9 months) had a 42% significantly decreased risk of death (HR = 0.58, 95% CI: 0.35–0.97), compared with those without diabetes (MST, 42.0 months).

**Table 1:** Patient characteristic and clinical features.

Variables	Patients	Deaths	MST (mo)	Log-rank *P*	HR (95% CI)
	n = 576 (%)	n = 206			
Age				0.350	
< = 60	273 (47.4)	92	53.2^a^		1.00
>60	303 (52.6)	114	42.0		1.14 (0.87–1.50)
Sex				0.002	
male	380 (66.0)	147	39.0		1.00
female	196 (34.0)	59	55.0		0.63 (0.46–0.85)
Smoking status				<0.001	
never	309 (53.6)	94	55.0		1.00
ever	267 (46.4)	112	32.0		1.76 (1.34–2.32)
Diabetes mellitus				0.034	
none	508 (88.2)	190	42.0		1.00
yes	68 (11.8)	16	54.9^a^		0.58 (0.35–0.97)
Histology				0.071	
adenocarcinoma	381 (66.2)	127	55.0		1.00
squamous Cell	166 (28.8)	71	36.0		1.40 (1.05–1.87)
others^b^	29 (5.0)	8	40.8^a^		1.07 (0.52–2.18)
Clinical stage				<0.001	
I	104 (18.1)	21	65.4^a^		1.00
II	97 (16.8)	32	55.0		1.96 (1.13–3.41)
III	102 (17.7)	38	39.0		2.51 (1.47–4.28)
IV	273 (47.4)	115	31.0		3.69 (2.31–5.90)
Surgical operation				<0.001	
none	326 (56.6)	140	26.0		1.00
yes	250 (43.4)	66	58.0^a^		0.37 (0.27–0.50)
Chemotherapy				0.079	
none	65 (9.5)	26	31.5		1.00
yes	521 (90.5)	180	43.0		1.44 (0.95–2.18)
Targeted therapy				0.058	
none	480 (83.3)	174	46.0		1.00
yes	96 (16.7)	32	42.0		0.70 (0.48–1.02)
SETD8 rs16917496					
Codominant model				0.006	
TT	282 (49.0)	106	41.0		1.00
CT	234 (40.6)	84	47.3		0.85 (0.64–1.13)
CC	60 (10.4)	16	58.0		0.44 (0.26–0.74)
*P* for trend				0.003	0.73(0.60–0.90)
Dominant model				0.031	
TT	282 (49.0)	106	41.0		1.00
CT+CC	294 (51.0)	100	52.8		0.74 (0.56–0.98)
Recessive model				0.003	
TT+CT	516 (89.6)	190	41.0		1.00
CC	60 (10.4)	16	58.0		0.47 (0.28–0.79)

a Mean survival time was provided when MST could not be calculated.

b Other carcinomas include large cell, undifferentiated and mixed-cell carcinomas.

### Effect of the SET8 3′UTR rs16917496 T>C Polymorphism on NSCLC Survival

**Figure 2:**
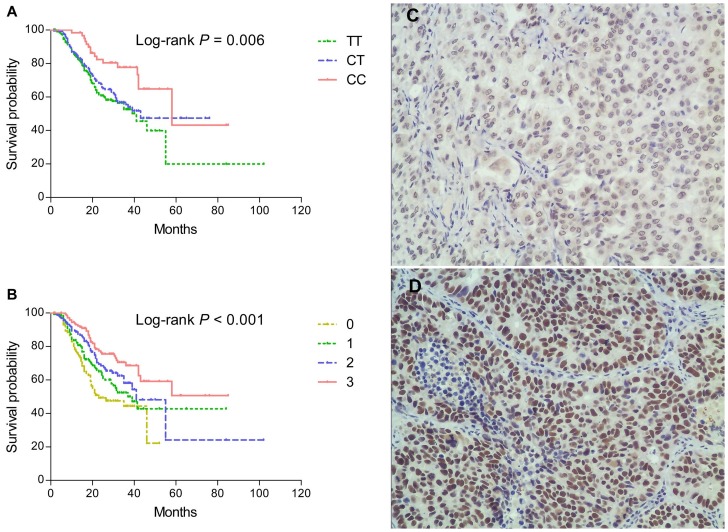
Kaplan-Meier plots of survival by *SET8* genotypes in NSCLC patients’ survival and SET8 protein expression levels in NSCLC tissues with immunohistochemistry. (A) *SET*8 rs16917496 genotype and NSCLC survival (log-rank *P* = 0.006) in a codominant model. (B) Kaplan-Meier plots of survival by combination of *SET8* genotypes and smoking status in NSCLC survival (log-rank *P*<0.001). 0, patients with common genotype (TT) and ever smoking; 1, those with variant genotypes (CT or CC) and ever smoking; 2, those with common genotype but without smoking; 3, those with variant genotypes and without smoking. (C) low expression; (D) high expression. Cells with a brown-stained nucleus are regarded as positive. Original magnification: ×200. NSCLC, non-small cell lung cancer.

The genotype frequencies of the rs16917496 were in Hardy-Weinberg equilibrium (*P* = 0.272). Consistent with previous reports, the C allele was found to be the minor frequency allele [21], [35], [36], [37]. Log-rank test detected a significant association of the rs16917496 with NSCLC survival in different genetic models (*P* = 0.006, 0.031 and 0.003 for codominant model, dominant model and recessive model, respectively. Figure 2A). Patients carrying the rs16917496 CC genotype had an improved OS compared to those with TT genotype (58.0 vs. 41.0 months, HR = 0.44, 95% CI: 0.26–0.74). Univariate Cox regression analysis showed that this SNP was a significant prognostic marker of NSCLC (dominant model: HR = 0.74, 95% CI: 0.56–0.98; recessive model: HR = 0.47, 95% CI: 0.28–0.79) (Table 1). The association remain significant after adjusted for age, sex, smoking status, diabetes mellitus, histology, clinical stage, surgical operation and treatment status (dominant model: HR = 0.70, 95% CI: 0.53–0.92). Moreover, a study power of 91.7% (two-sided test, α = 0.05) has been achieved to detect an HR of 0.70 for the C allele genotypes in the dominant model.

In order to find independent prognostic factors, we further did a multivariate stepwise Cox regression analysis with selected demographic characteristics, clinical features and the SET8 genotype on NSCLC survival. The results indicated that the *SET8* rs16917496 polymorphism (*P* = 0.014) was remained in the final predictive model together with smoking status, diabetes mellitus and surgical operation (*P* = 0.006, 0.018 and <0.001, respectively) (Table 2).

**Table 2:** Results of multivariate Cox regression analysis on NSCLC patients’ survival.

Variables	β	SE	HR	95% CI	*P* value
Stepwise regression analysis					
Sex (female vs. male )	−0.254	0.211	0.78	0.78 (0.51–1.17)	0.228
Smoking status(ever vs. never)	0.523	0.191	1.69	1.69 (1.16–2.46)	0.006
Diabetes mellitus(yes vs. no)	−0.619	0.261	0.54	0.54 (0.32–0.90)	0.018
Clinical stage(III–IV vs. I–II)	0.203	0.247	1.23	1.23 (0.78–1.99)	0.411
Surgical operation(yes vs. no)	−0.935	0.232	0.39	0.39 (0.25–0.62)	<0.001
Rs16917496(CT+ CC vs. TT)	−0.345	0.140	0.71	0.71 (0.54–0.93)	0.014

### Stratification and Interaction Analysis

The association between *SET8* rs16917496 polymorphism and NSCLC survival was further evaluated by stratified analysis of smoking status, diabetes mellitus, histology, clinical stage, surgical operation, chemotherapy and targeted therapy status. As shown in Table 3, the protective effect of variant genotypes of *SET8* rs16917496 were more prominent in never smokers (adjusted HR = 0.54, 95% CI: 0.35–0.83), patients without diabetes (adjusted HR = 0.70, 95% CI: 0.53–0.94), patients who received chemotherapy (adjusted HR = 0.69, 95% CI: 0.51–0.92) but not targeted therapy (adjusted HR = 0.52, 95% CI: 0.24–1.02). Heterogeneity test showed that heterogeneity in every two strata were significant for smoking status (*P* = 0.016). Therefore, a gene-smoking status interaction analysis was carried out (Table 4), and there was a statistically significant multiplicative interaction between the genotypes of rs16917496 and smoking status on NSCLC survival (*P* for multiplicative interaction <0.001). Compared to smokers with rs16917496 TT genotype, never smokers with CC+CT genotypes had a significantly decreased risk of death (adjusted HR = 0.50, 95% CI: 0.25–0.64). Kaplan-Meier plots of survival by combination of *SET8* genotypes and smoking status in NSCLC-specific survival are shown in Figure 2B.

**Table 3:** Stratified analysis for rs16917496 genotypes and NSCLC patients’ survival.

Variables	Rs16917496 deaths/patients		Adjusted HR (95% CI)^a^	*P* value	Heterogeneity *P*
	TT	CT+CC			
Total	106/282	100/294	0.70 (0.53–0.92)	0.011	
Smoking status					0.016
never	54/162	40/147	0.54 (0.35–0.83)	0.006	
ever	52/120	60/147	0.92 (0.63–1.36)	0.685	
Diabetes mellitus					0.484
none	97/246	93/262	0.70 (0.53–0.94)	0.017	
yes	9/36	7/32	0.52 (0.13–2.05)	0.354	
Histology					0.277
adenocarcinoma	69/191	58/190	0.65 (0.46–0.93)	0.018	
squamous Cell	34/74	37/92	0.60 (0.36–0.98)	0.039	
others^b^	3/17	5/12	–	0.807	
Clinical stage					0.051
I	7/44	14/60	1.22 (0.44–3.41)	0.689	
II	18/53	14/44	0.48 (0.22–1.03)	0.058	
III	18/41	20/61	0.56 (0.27–1.17)	0.123	
IV	63/144	52/129	0.75 (0.51–1.09)	0.131	
Surgical operation					0.687
none	73/162	67/164	0.72 (0.51–1.01)	0.060	
yes	33/120	33/130	0.65 (0.39–1.08)	0.098	
Chemotherapy					0.406
none	10–24	16/31	1.24 (0.47–3.24)	0.663	
yes	96/258	84/263	0.69 (0.51–0.92)	0.013	
Targeted therapy					0.502
none	89/238	85/242	0.70 (0.52–0.95)	0.023	
yes	17/44	15/52	0.53 (0.24–1.20)	0.129	

a Adjusted for age, sex, smoking status, diabetes mellitus, histology, clinical stage, surgical operation and treatment status.

b Other carcinomas include large cell, undifferentiated and mixed-cell carcinomas.

**Table 4:** Interaction analysis between rs16917496 genotypes and smoking status.

Variables	Patients	Deaths	MST (months)	Crude HR (95% CI)	Adjusted HR (95% CI)^a^
rs16917496 genotypes and smoking status					
TT with ever smoking	120	52	23.0	1.00	1.00
CT+CC with ever smoking	147	60	39.0	0.72(0.50–1.05)	0.84(0.57–1.23)
TT with never smoking	162	54	41.0	0.57(0.39–0.83)	0.71(0.44–1.16)
CT+CC with never smoking	147	40	58.2^b^	0.38(0.25–0.38)	0.40(0.25–0.64)
*P* for multiplicative interaction				<0.001	<0.001

a Adjusted for age, sex, diabetes mellitus, histology, clinical stage, surgical operation and treatment status.

b Mean survival time was provided when MST could not be calculated.

### Effect of the SET8 3′UTR rs16917496T>C Polymorphism on SET8 Expression

The rs16917496 T>C polymorphism located at the binding site of miR-502 (Figure 1A). As predicted using RNAhybrid [39], miR-502 has a higher minimum free energy (MFE) with C allele (|MFE| = 16.2 kcal/mol) of rs16917496 in *SET8* than that with T allele (|MFE| = 14.5 kcal/mol). Thus, we hypothesized the variant C allele might lead to a reduced expression of *SET8* resulted from increased miRNA repression. To test this hypothesis, two luciferase reporter gene constructs contained rs16917496 T or C allele were generated to determine whether this SNP could affect gene expression (Figure 1C). The transcription activity of reporter gene with rs16917496 C allele was significantly lower as compared with T allele when we co-transfected chemically synthesized mature miR-502 into A549 cell (*P*<0.0001) and 293T cell (*P* = 0.002). The miR-502 inhibitors could significantly reverse the activities of reporter gene with the rs16917496 C allele (*P*<0.0001 for both A549 and 293T); however, no evident change was observed for reporter gene with T allele treated with miR-502 inhibitors (*P*>0.05 for both). Taken together, the strong effect of miR-502 on modulating *SET8* in both A549 and 293T cell lines indicated that the miR-502 specially binds to the 3′UTR of *SET8* gene with rs16917496 C allele and suppress the expression of the *SET8* gene *in vitro*.

### Association of the SET8 Protein Expression with rs16917496 T>C Polymorphism and NSCLC Survival

Among the 576 NSCLC blood samples, 192 had sufficient matching formalin-fixed, paraffin-embedded lung cancer specimens. We then explored the expression status of SET8 in NSCLC and the association with rs16917496 polymorphism using immunohistochemistry (Figure 2C and 2D). SET8 was highly expressed in 50.5% lung cancer tissues. Patients with the *SET8* CC genotype had significantly lower levels of SET8 expression than did those with the CT or TT genotype (*P* = 0.007, Table S1), which confirmed the results of luciferase reporter assays. These results supported a genotype-phenotype relation that the rs16917496 CC variant genotype confers to a lower expression of SET8 gene compared with CT or TT genotypes. The survival rate of NSCLC patients with low and high SET8 expression levels was further examined using log-rank test. Individuals with low levels of SET8 displayed longer OS than those with high levels of SET8 (55.0 vs. 43.1 months), although the difference was not statistically significant (*P* = 0.138).

## Discussion

MicroRNAs are a new class of non-coding RNAs and have been shown to play an important role in regulating protein-coding genes. Emerging evidence has suggested that polymorphisms within miRNA-binding sites may affect the miRNA regulation to target gene expression and consequently modify cancer risk and outcome. For instance, the variant A allele of *RAP1A* rs6573 enhanced the binding ability of miR-196a, leading to an increased miRNA-mediated *RAP1A* repression, and this SNP functioned as a potential personal diagnostic marker for esophageal squamous cell carcinoma [40]. A SNP (rs13312986) in miRNA-629 binding site altered the *NBS1* expression and contributed to lung cancer risk [41]. Allelic variation of rs3134615 might destroy the capacity of miR-1827 to regulate *MYCL1* expression and this variant was associated with small cell lung cancer risk [42]. Rs7180135 is located within the miR-197 binding site in the 3′UTR of *RAD51*, and the minor allele was reported to be associated with an improved cancer-specific survival of bladder cancer patients [43]. In this study, we examined a SNP in the miR-502 binding site of the *SET8* 3′UTR for its predictive power related to NSCLC outcomes. We showed that the rs16917496 T>C was associated with NSCLC survival in a Chinese population. The variant allele C may decrease the expression of *SET8* through enhancing the binding capacity of miR-502 to target site in the 3′UTR of *SET8*. The CC genotype was associated with reduced SET8 protein expression, which was consistent with previous studies in breast cancer had hepatocellular carcinoma [35], [37]. Moreover, lower levels of SET8 were associated with a longer survival in NSCLC.

SET8 is found to be overexpressed in various types of tumor, including lung cancer [44]. The function of SET8 is likely to be very broad and it has been implicated in pathological processes such as tumorigenesis. It has been reported that SET8 is required for normal S-phase progression [26], [29], is engaged in transcriptional regulation [24], [25], genome replication and stability [26], [27], [28], and modulates the proapaptotic and cell-cycle arrest functions [30], [31], [33]. SET8 has a well-defined function in the TP53 pathway by monomethylating p53 at lysine 382 and suppressing the p53-mediated transcription activation of target genes [33]. The most notable function of SET8 is the modulation of chromatin dynamics as a histone-modifying enzyme [45]. It is well established that epigenetic alterations of the histone code contribute to the initiation of multiple malignancies, such as lymphomas, squamous cell carcinoma and colorectal adenocarcinoma [46], [47]. These epigenetic changes appear at an early stage of carcinogenesis and accumulate during progression [47]. Recently, Takawa et al. [44] revealed a function of SET8 for lysine methylation on a non-histone protein PCNA, which functions are related to vital cellular processes. SET8 has been found to promote carcinogenesis by deregulating PCNA expression. Meanwhile, a novel role for SET8 in tumor invasion and metastasis was established by Yang et al. [34]. They demonstrated that SET8 promotes epithelial-mesenchymal transition (EMT) and enhances the invasive capacity of breast cancer cells via functional interdependence with a transcription factor TWIST and through dual chromatin remodelling activity. Taken together, SET8 plays an important role in the development and progression of cancer.

The expression status and function role of miR-502 in lung cancer is largely unknown. However, miR-502 was found to be downregulated in colon cancer specimens compared with the paired normal control samples. Ectopic expression of miR-502 inhibited autophagy, cell growth and cell-cycle progression of colon cancer cells in vitro. MiR-502 also inhibited colon cancer growth in a mouse tumor xenografts model [48]. The miR-502 binding site SNP rs16917496 in the 3′UTR of *SET8*, first identified by Yu et al. [21], has been reported to contribute to susceptibility of breast cancer and ovarian cancer [35], [36], and clinical outcome of small cell lung cancer and hepatocellular carcinoma [37], [49]. According to *in silico* analysis using RNAhybrid database, miR-502 is predicted to strongly bind with the target site of *SET8* harboring C allele of rs16917496. Luciferase assay indicated that the transcription activity of reporter gene with rs16917496 C allele was significantly decreased than that with T allele. The downregulated level of SET8 might result in an inhibitor to tumorigenesis and progression. This was consistent with the association results that the C allele of rs16917496 was associated with a better prognosis of NSCLC. Further in-depth functional studies are required to uncover the exact mechanism of this variant.

It is well studied that smoking is a strong risk factor of lung cancer. We also found it to be an unfavorable prognostic factor for NSCLC patients. Carcinogens in cigarettes can cause DNA damage, which may lead to overexpression of p53 in primary lung cancer [50] and downregulation of SET8 expression [33]. SET8 modulates p53 expression by methylating p53 at lysine 382. Depletion of SET8 augments the proapoptotic and checkpoint activation functions of p53 [33]. Meanwhile, the rs16917496 C allele may decrease the expression of *SET8* through enhancing the binding capacity of miR-502 to target the 3′UTR of *SET8*. In the present study, a significant interaction was observed between rs16917496 and smoking status. Non-smoking patients carrying at least one C allele of rs16917496 have a significantly longer OS than smokers or those with TT genotypes. It is plausible that genetic variations in *SET8* gene may modify the development of lung cancer mediated by smoking status.

Besides, there were still some limitations in this study. Firstly, only one potential functional SNP of *SET8* gene were investigated, which did not cover all variants of *SET8* and restricted further haplotype analysis. Secondly, our study was based on a relative small sample size. Although we observed a significant association between rs16917496 polymorphism and NSCLC survival and an interaction effect between this SNP and smoking status. Biological assays have demonstrated that rs16917496 is biological functional. Therefore, it supported that our finding that rs16917496 CC variant genotype associated with a reduced risk of death for NSCLC is unlikely to be achieved by chance.

In summary, we confirmed that *SET8* 3′UTR rs16917496 T>C polymorphism might predict NSCLC patients’ survival in a Chinese population. A functional assay suggested that the genetic variation rs16917496 in the miR-502 binding site could modify NSCLC outcome through regulating the expression of *SET8*. The findings further highlight that polymorphisms in miRNA-binding sites may play an important role in lung cancer and may have an effect on patients’ clinical outcome.

## Supporting Information

Table S1 **Correlation of rs16917496 genotype and SET8 expression level.**
(DOC)
