# Supplementary material for: Genetic Variation in a MicroRNA-502 Minding Site in SET8 Gene Confers Clinical Outcome of Non-Small Cell Lung Cancer in a Chinese Population
Source: PLoS One. 2013 Oct 11;8(10):e77024. doi: 10.1371/journal.pone.0077024 (PMC3795636; doi:10.1371/journal.pone.0077024)
Supplement: Table S1 — Correlation of rs16917496 genotype and SET8 expression level. (DOC) [file pone.0077024.s001.doc]

**Table S1.** Correlation of rs16917496 genotype and SET8 expression level.

| Expression level |  | genotype |  |  |
| --- | --- | --- | --- | --- |
|  | TT | CT | CC | *P*^a^ |
|  |  |  |  | 0.007 |
| High | 49 | 44 | 4 |  |
| Low | 34 | 45 | 16 |  |

^a^χ^2^ test for genotype distributions between high and low expression levels of SET8 protein.
